# Supplementary material for: Network analysis of regional livestock trade in West Africa
Source: PLoS One. 2020 May 14;15(5):e0232681. doi: 10.1371/journal.pone.0232681 (PMC7224501; doi:10.1371/journal.pone.0232681)
Supplement: S1 Table — (DOCX) [file pone.0232681.s001.docx]

**S1 Table. Percentage of livestock movements by type 2013-2017.** Movements in the data disaggregated by movement, transport and livestock type. Rows show the first type and the column the second, and the main diagonal shows the proportion of all movements belonging to each category within each classification (by movement type, by transport type and by livestock type). For example, of all on-the-hoof movements, 73.3% transported cattle and 20.0% sheep, whereas of all movements 6% concerned goats. 59.9% of all cattle movements were international while the rest did not cross borders.


|  | By mov type | | By transport type | | | By livestock type | | | |
| --- | --- | --- | --- | --- | --- | --- | --- | --- | --- |
|  | Int'l | National | On the hoof | Train | Vehicle | Cattle | Donkey | Goat | Sheep |
| Int'l | **59%** |  | 4% | 1% | 95% | 75% | 0% | 8% | 17% |
| National |  | **41%** | 4% | 1% | 94% | 72% | 0% | 3% | 25% |
| On the hoof | 60% | 40% | **4%** |  |  | 73% | 0% | 7% | 20% |
| Train | 49% | 51% |  | **1%** |  | 68% | 0% | 4% | 27% |
| Vehicle | 59% | 41% |  |  | **94%** | 74% | 0% | 6% | 20% |
| Cattle | 60% | 40% | 4% | 1% | 94% | **74%** |  |  |  |
| Donkey | 100% |  |  |  | 100% |  | **0%** |  |  |
| Goat | 79% | 21% | 5% | 1% | 94% |  |  | **6%** |  |
| Sheep | 50% | 50% | 4% | 2% | 94% |  |  |  | **20%** |
