# Supplementary material for: Network analysis of regional livestock trade in West Africa
Source: PLoS One. 2020 May 14;15(5):e0232681. doi: 10.1371/journal.pone.0232681 (PMC7224501; doi:10.1371/journal.pone.0232681)
Supplement: S2 Table — (DOCX) [file pone.0232681.s002.docx]

**S2 Table. Fitting the degree distribution.** The procedure suggested by Clauset et al. was implemented using the poweRlaw package in R. Initial testing suggested the powerlaw and log-normal distributions were the two most plausible ones (exponential and Poisson were also tested). The KS statistics indicated that the power law was plausible for the movement sequence. Both the power law and log-normal distributions were plausible, but the likelihood ratio did not favor one or the other because of our limited sample size. The hypothesis p-values were drawn from 1000 bootstrapping iterations in each case.

| **Degrees** | **Movements (A)** | | **Links (B)** | |
| --- | --- | --- | --- | --- |
| **Distribution** | **Power law** | **Log-normal** | **Power law** | **Log-normal** |
| **x_min_** | 84 | 4 | 5 | 4 |
| **Parameter estimate(s)** | 1.659 | 3.571, 2.343 | 2.152 | -2.410, 0.036 |
| **Kolmogorov-Smirnoff (KS) Statistic** | 0.064 | 0.048 | 0.038 | 0.037 |
| **Hypothesis p-value^a^** | 0.400* | 0.050 | 0.690* | 0.450* |
| **Likelihood ratio (R)^b^** | -0.961 (x_min_=84) | | -0.515 (x_min_=5) | |
| **Likelihood ratio p-value** | 0.832 | | 0.697 | |

^a^Following Clauset et al., we consider the fit as plausible if p>0.10.

^b^Vuong’ test likelihood ratio (R)

*Plausible fit
