# Supplementary material for: Network analysis of regional livestock trade in West Africa
Source: PLoS One. 2020 May 14;15(5):e0232681. doi: 10.1371/journal.pone.0232681 (PMC7224501; doi:10.1371/journal.pone.0232681)

**S1 Fig. Proportion of movements by type of movement and month 2013-2017.** White dashed lines indicate Tabaski (a religious festival) dates for each year.


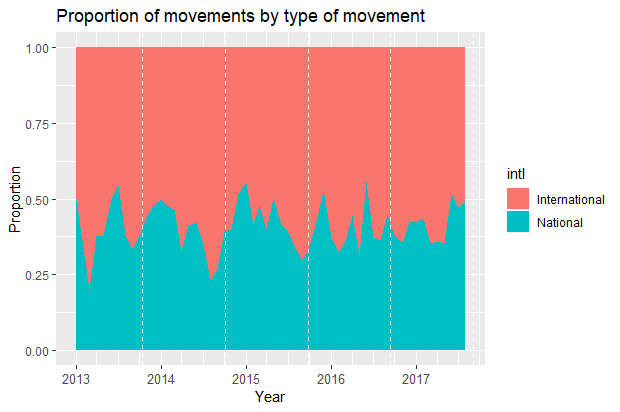

Supplement: S1 Fig — (DOCX) [file pone.0232681.s003.docx]
