# Supplementary material for: Network analysis of regional livestock trade in West Africa
Source: PLoS One. 2020 May 14;15(5):e0232681. doi: 10.1371/journal.pone.0232681 (PMC7224501; doi:10.1371/journal.pone.0232681)

**S2 Fig. Proportion of movements by type of livestock and month 2013-2017.** White dashed lines indicate Tabaski dates for each year. Sheep movement proportions fluctuate throughout each year, being at their maximum in the months preceding Tabaski.


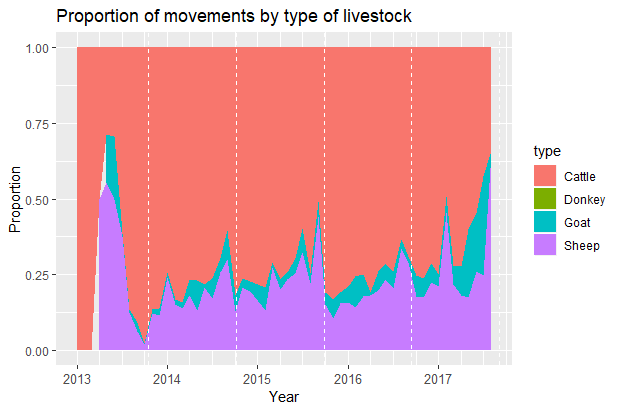

Supplement: S2 Fig — (DOCX) [file pone.0232681.s004.docx]
