# Supplementary material for: Network analysis of regional livestock trade in West Africa
Source: PLoS One. 2020 May 14;15(5):e0232681. doi: 10.1371/journal.pone.0232681 (PMC7224501; doi:10.1371/journal.pone.0232681)

**S3 Fig. Proportion of movements by type of transport and month 2013-2017.** White dashed lines indicate Tabaski dates for each year. Most movements in the data were made by vehicle, with smaller proportions being made on the hoof and by train. In general, vehicle movements increase in months preceding Tabaski.


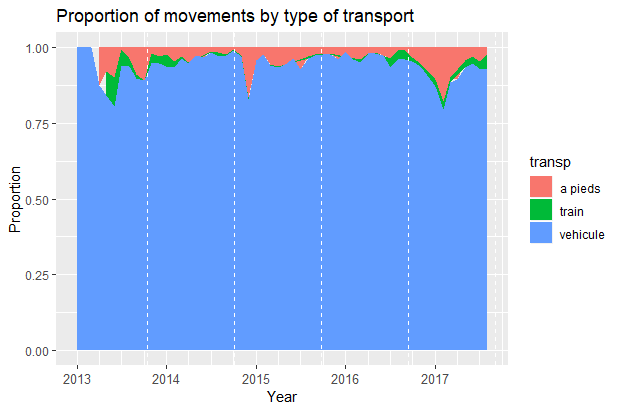

Supplement: S3 Fig — (DOCX) [file pone.0232681.s005.docx]
