# Supplementary material for: Network analysis of regional livestock trade in West Africa
Source: PLoS One. 2020 May 14;15(5):e0232681. doi: 10.1371/journal.pone.0232681 (PMC7224501; doi:10.1371/journal.pone.0232681)
Supplement: S1 File — (DOCX) [file pone.0232681.s006.docx]

**S1 File. Data collection methodology.**

USAID’s Agricultural Trade Promotion (ATP) and Extended-ATP (E-ATP) projects collected movement data for selected agricultural commodities in West Africa to track the direction and magnitude of intraregional trade. Data was collected in selected markets and border crossing points on major trade corridors between Benin, Burkina Faso, Côte d’Ivoire, Ghana, Mali, Nigeria, Senegal and Togo. Markets and exit points are monitored daily or on market days, and monitored products included ruminant livestock (cattle, sheep, goats and recently donkeys), cereals and grains (maize, millet, sorghum and rice). After the ATP and E-ATP projects ended in 2013, data collection was adopted by the CILSS under their Regional Program for Market Access (Programme Régionaux d’Accès aux Marchés, PRA-Marchés) starting in May 2013. Before collection was transferred to CILSS, origin and destination points of the shipments (called loading and unloading points in the database) were not recorded.

This supplementary information file intends to briefly describe and familiarize the reader with the data collection methodology. The reader is referred to the official documentation for further details and updated procedures (available from [www.agrictrade.net](http://www.agrictrade.net)). It is possible that by the time of publication of this manuscript, changes are made to the data collection methodology.

Data collection is carried out with the support of partner organizations throughout the region. Groups of data collectors are assigned to a focal point (person) in each country. The focal point is in charge of collating survey data and transferring the data to CILSS monthly. The focal point supervises the enumerators in the field, checks completed data collection forms, and cross-checks average prices of commodities. The focal point also identifies problems with collected data. A list of current focal points can be found in <https://agrictrade.net/focal-points/>.

The livestock data pipeline is as follows:

1. Two experienced, trained enumerators are sent to each data collection point
2. Both data collectors complete the CILSS data collection forms, that include:
   - Date of flow
   - Mean of transport: vehicle or on foot
   - Vehicle number (license plate)
   - Origin country
   - Loading point (in origin country)
   - Collection point (data collection)
   - Survey point
   - Type of animal: cattle, goat, sheep, donkey
   - Number of big animals
   - Unit price per big animal
   - Number of medium animals
   - Unit price per medium animal
   - Number of small animals
   - Unit price per small animal
   - Number of total heads
   - Transport cost
   - Value in local currency
   - Destination country
   - Unloading point (in destination country)
3. At the end of the day, data recorded manually by both collectors are compared and adjustments are made, if necessary
4. Information from the data collection forms are recorded in a register; if completed forms are lost or damaged, information can be retrieved from this register
5. At the end of each month, accumulated forms are transferred by enumerators to the corresponding focal point
6. Information is entered into a spreadsheet by the focal point, then transferred to the CILSS electronically early in the following month
7. Once available to CILSS, data from all focal points are agglomerated in one spreadsheet, and processed
8. Findings (volumes and values, changes) are reported monthly, quarterly or annually by the CILSS and/or the focal points.

Some of the challenges faced by the data collection directly affect the quality of the data. These include:

- Incomplete data. Market conditions can affect complete data collection. For example, it is possible that some movements go unrecorded during high-volume days. Additionally, the origin and final destination of the animals might not be known by the traders.
- Security conditions. Some markets might be unsafe to survey when security conditions deteriorate (for example, collection points in northern Nigeria).
- Lack of variable value standardization. Data is entered manually and not coded by the enumerators. For example, many versions of a single location name exist.
